# NMR chemical shift pattern changed by ammonium sulfate precipitation in cyanobacterial phytochrome Cph1

**DOI:** 10.3389/fmolb.2015.00042

**Published:** 2015-07-28

**Authors:** Chen Song, Christina Lang, Jakub Kopycki, Jon Hughes, Jörg Matysik

**Affiliations:** ^1^Leids Instituut voor Chemisch Onderzoek, Universiteit LeidenLeiden, Netherlands; ^2^Institut für Analytische Chemie, Fakultät für Chemie and Mineralogie, Universität LeipzigLeipzig, Germany; ^3^Institut für Pflanzenphysiologie, Justus-Liebig-Universität GießenGießen, Germany

**Keywords:** biliprotein, photoreceptor, phycocyanobilin, red-absorbing state, dehydration process, solid-state NMR

## Abstract

Phytochromes are dimeric biliprotein photoreceptors exhibiting characteristic red/far-red photocycles. Full-length cyanobacterial phytochrome Cph1 from *Synechocystis* 6803 is soluble initially but tends to aggregate in a concentration-dependent manner, hampering attempts to solve the structure using NMR and crystallization methods. Otherwise, the Cph1 sensory module (Cph1Δ2), photochemically indistinguishable from the native protein and used extensively in structural and other studies, can be purified to homogeneity in >10 mg amounts at mM concentrations quite easily. Bulk precipitation of full-length Cph1 by ammonium sulfate (AmS) was expected to allow us to produce samples for solid-state magic-angle spinning (MAS) NMR from dilute solutions before significant aggregation began. It was not clear, however, what effects the process of partial dehydration might have on the molecular structure. Here we test this by running solid-state MAS NMR experiments on AmS-precipitated Cph1Δ2 in its red-absorbing Pr state carrying uniformly ^13^C/^15^N-labeled phycocyanobilin (PCB) chromophore. 2D ^13^C–^13^C correlation experiments allowed a complete assignment of ^13^C responses of the chromophore. Upon precipitation, ^13^C chemical shifts for most of PCB carbons move upfield, in which we found major changes for C4 and C6 atoms associated with the *A*-ring positioning. Further, the broad spectral lines seen in the AmS ^13^C spectrum reflect primarily the extensive inhomogeneous broadening presumably due to an increase in the distribution of conformational states in the protein, in which less free water is available to partake in the hydration shells. Our data suggest that the effect of dehydration process indeed leads to changes of electronic structure of the bilin chromophore and a decrease in its mobility within the binding pocket, but not restricted to the protein surface. The extent of the changes induced differs from the freezing process of the solution samples routinely used in previous MAS NMR and crystallographic studies. AmS precipitation might nevertheless provide useful protein structure/functional information for full-length Cph1 in cases where neither X-ray crystallography nor conventional NMR methods are available.

## Introduction

Phytochromes modulate various biological responses to light in almost all phases of plant development (Franklin and Quail, 2010). Plant phytochromes represent a paradigm for a large and diverse set of photochromic photoreceptors that are also known in many microorganisms (Kehoe and Grossman, 1996; Hughes et al., 1997; Yeh et al., 1997; Davis et al., 1999; Bhoo et al., 2001; Giraud et al., 2002; Froehlich et al., 2005; De Riso et al., 2009; Rockwell et al., 2014b). Phytochromes typically photoconvert between red-absorbing (Pr) and far-red-absorbing (Pfr) states via a C15-*Z*/*E* isomerization of their covalently bound linear tetrapyrrole (bilin) chromophores (Rockwell et al., 2006; Hughes, 2010; Rockwell and Lagarias, 2010; Song et al., 2011a; Yang et al., 2011). The bilin chromophore such as phycocyanobilin (PCB), phytochromobilin (PΦB), or biliverdin (BV) is buried in a conserved pocket formed in the GAF (c*G*MP phosphodiesterase, *a*denylate cyclase, *F*hlA) domain which is part of a knotted N-terminal photosensory module also comprising PAS (*P*eriod/*A*RNT/*S*ingle-minded) and PHY (*phy*tochrome-specific) domains. The tripartite sensory module is conserved in canonical phytochromes and bacteriophytochromes (Wagner et al., 2005; Essen et al., 2008; Yang et al., 2008; Nagatani, 2010; Auldridge and Forest, 2011; Burgie et al., 2014). Phytochromes as well as a large group of related photoswitchable biliproteins, the cyanobacteriochromes (CBCRs) (Rockwell et al., 2011, 2012, 2014a; Chen et al., 2012; Hirose et al., 2013; Narikawa et al., 2013) have attracted increasing attention as *in vivo* fluorophores, in optogenetics and in synthetic biology (Shimizu-Sato et al., 2002; Levskaya et al., 2005, 2009; Shu et al., 2009; Tabor et al., 2009; Möglich and Moffat, 2010; Zhang et al., 2010; Lee et al., 2013; Müller and Weber, 2013; Müller et al., 2013, 2014a,b; Piatkevich et al., 2013; Tischer and Weiner, 2014; Yu et al., 2014; Ziegler and Möglich, 2015). Phytochrome absorbance and fluorescence at red to near-infrared wavelengths makes the superfamily interesting for studies involving whole, living organisms whose tissues scatter light particularly strongly at short wavelengths. Even more valuable is their peculiar photochromicity whereby two (meta)stable ground states with different absorbance maxima and physiological activities exist, thereby allowing biological processes to be switched on or off by a brief pulse of red or far-red light (Borthwick et al., 1952). Neither the photochromic absorbance properties nor its mechanistic connection to signaling are well understood, however.

Cyanobacterial phytochrome Cph1 from *Synechocystis* 6803 (Hughes et al., 1997; Yeh et al., 1997) represents the evolutionary link between bacteriophytochromes and plant phytochromes. The complete chromophore-bearing sensory module (amino acids 1–515) of the full-length holoCph1 (termed Cph1Δ2) exhibits almost identical absorption spectra (van Thor et al., 2001) and photodynamics (Sineshchekov et al., 2002) to those of the native molecule. Cph1Δ2 has proven to be an especially useful model for basic studies of structure/function relationship in full-length protein because of its tractability for the recombinant expression in *Escherichia coli* cells (Lamparter et al., 1997) and availability for biophysical studies including X-ray crystallography (Essen et al., 2008; Mailliet et al., 2009) as well as both liquid-state (Strauss et al., 2005a; van Thor et al., 2006; Hahn et al., 2008) and solid-state (Rohmer et al., 2006, 2008, 2010a; Song et al., 2011a,b) NMR spectroscopy. These studies together with other physical and spectroscopic methods (van Thor et al., 2001, 2005; Dasgupta et al., 2009; Mroginski et al., 2009; Rockwell et al., 2009; Kim et al., 2012b, 2013, 2014a,b; Yang et al., 2012; Velazquez Escobar et al., 2015), and mutagenesis (Fischer and Lagarias, 2004; Strauss et al., 2005b; Hahn et al., 2006) have provided an improved mechanistic understanding of the phytochrome photosensor.

Cph1Δ2 lacks the C-terminal transmitter module, comprising an ATP-binding/kinase domain as well as an amphiphilic helix-loop-helix largely responsible for dimerization (Matsushita et al., 2003; Mateos et al., 2006) and a histidine phosphoacceptor proximal to the sensory module. Since there is no 3D structure of the complete structure of any phytochrome available (see Essen et al., 2008; Yang et al., 2008, 2009; Scheerer et al., 2010; Burgie et al., 2014; Takala et al., 2014), how the light signal is propagated from the bilin through the sensor module to affect the kinase/phosphotransferase activities of the transmitter module remains conjectural. Domain-swapping experiments with Cph1 and EnvZ, however, imply that the mechanism of intramolecular signaling in SHPK's (sensory histidine protein kinases) is conserved (Levskaya et al., 2005).

Attempts to solve the 3D structure of full-length holoCph1 have been impeded by concentration-dependent aggregation in solution. Psakis et al. (2011) found that glycerol/xylitol retards aggregation significantly, but does not prevent it. The problems associated with aggregation might be circumvented by precipitating the material using ammonium sulfate (AmS) before it begins to aggregate, i.e., by collecting the SEC (size-exclusion chromatography) fractions directly into AmS. This procedure seems not to disturb the structure greatly, as the relative proportions of Pr and Pfr remain the same before and after AmS precipitation. Moreover, as previously described in full-length phyA phytochrome from *Avena sativa*, the low-temperature FTRR (Fourier-transform resonance Raman) spectra of AmS precipitates displayed similar vibrational band patterns to those of the frozen solutions (Matysik et al., 1995), implying that the effect of AmS precipitation is restricted to the hydration shell of the protein molecule. These relatively old FTRR data, however, would be insufficiently accurate to detect subtle modifications of the electronic structure of the bilin as well as its interactions with the direct binding pocket upon dehydration.

Previous MAS NMR studies on phytochromes used frozen solutions of highly concentrated proteins routinely, e.g., the complete sensory modules of Cph1 from *Synechocystis* 6803 and phyA3 from *A. sativa* (Rohmer et al., 2008, 2010a; Song et al., 2011a, 2012) as well as an isolated GAF-domain fragment of Cph2 from *Synechococcus* OS-B' (Song et al., 2014). Holoproteins of these phytochromes were produced by *in vitro* assembly with a uniformly ^13^C/^15^N-labeled PCB chromophore (*u*-[^13^C,^15^N]-PCB). Here, we use MAS NMR technique to assess whether the structure and immediate environment of the bilin in the *u*-[^13^C,^15^N]-PCB-holoCph1Δ2 are preserved in an AmS pellet using 2D ^13^C–^13^C dipolar correlation experiments which allow for a complete and unambiguous ^13^C assignment for the entire bilin. We find that almost all of bilin signals move upfield (smaller chemical shifts) in the precipitated sample relative to those obtained from the frozen solution. The global effect induced by the partial dehydration on the bilin electronic structure can be attributed predominantly to packing effects between the bilin and its binding pocket. It is, however, unlikely to arise from the local modification of bilin interaction with specific protein residues.

## Material and methods

### AmS precipitation of Cph1**Δ**2 as Pr

The *u*-[^13^C,^15^N]-PCB-holoCph1Δ2 was prepared and purified by Ni-affinity and SEC as described (Song et al., 2011a). Working in dim blue–green safelight (490 nm LED), 37.5 ml of cold AmS buffer (50 mM Tris; 3.3 M AmS; 1 mM IDA, pH 7.8) was added to 15 mg of this material in the Pr state (following saturating irradiation with 730 nm monochromatic red light) in 25 ml TESß (50 mM Tris; 5 mM EDTA; 300 mM NaCl; 1 mM β-mercaptoethanol, pH 7.8), gently mixed at 4 °C for >24 h. The precipitate was then pelleted at 10,000*g* for 10 min at 4 °C and most of the supernatant removed. Following centrifugation at 50,000*g*, the pellet was finally suspended in 600 μL of the original precipitant. This slurry was packed stepwise into a 4-mm zirconia MAS NMR rotor by centrifugation at 50,000*g*, the supernatant being removed after each spin. The final packed volume was 100 μL, comprising ca. 10 mg of holoprotein. The rotor was then snap-frozen in liquid nitrogen and kept at −80 °C.

### MAS NMR spectroscopy

**Figure 1:**
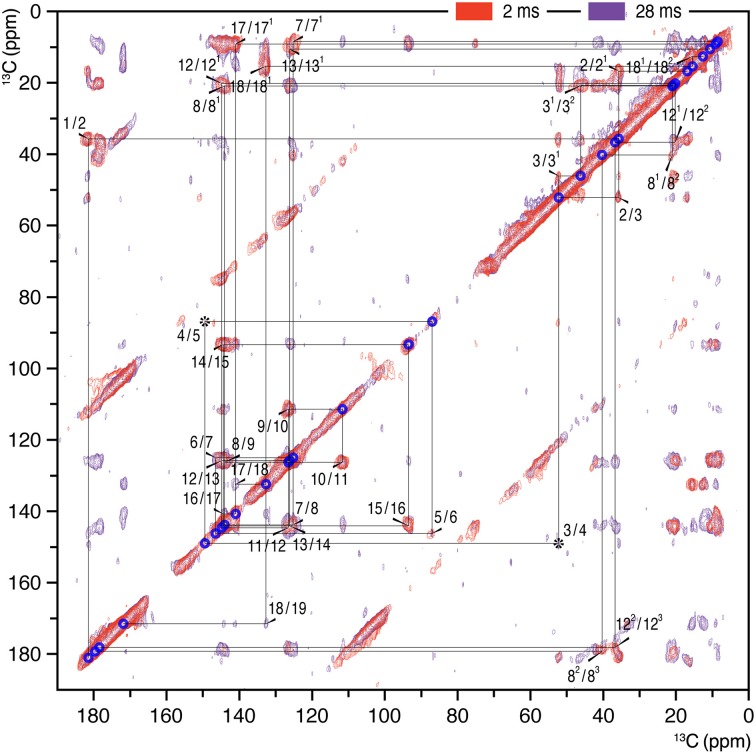
**2D ^13^C–^13^C homonuclear dipolar correlation spectra of AmS precipitated Cph1Δ2 holoprotein assembled with *u*-[^13^C,^15^N]-PCB chromophore in the Pr dark state**. Proton mixing times of 2 (red) and 28 ms (purple) were employed. The lines indicate sequences of nearest-neighbor correlations (for numbering, see **Figure 4**). The assignment of indirect-bonded correlation peaks is provided in Figure S1 (enlarged view of the DARR spectra with projections along both dimensions). The complete ^13^C chromophore assignment as Pr is listed in Table S1 and illustrated in **Figure 4**.

Two-dimensional ^13^C–^13^C dipolar-assisted rotational resonance (DARR) experiments were used to assign ^13^C chemical shifts of the *u*-[^13^C,^15^N]-PCB chromophore in Cph1Δ2 as an AmS pellet. The DARR spectra shown in Figure 1 and Figure S1 were recorded by using a DMX-400 spectrometer equipped with a 4-mm CP/MAS probe (Bruker, Karlsruhe). The rotor containing the *in vitro* assembled holo-Cph1Δ2 was cooled to −50 °C in the magnet. The DARR spectra were acquired at a MAS rate of 13 kHz with two mixing times of 2 and 28 ms for ^13^C homonuclear recoupling. During the mixing period, the ^1^H–^13^C dipolar interaction was recovered by ^1^H continuous wave irradiation with the intensity satisfying the *n* = 1 rotary-resonance condition (Takegoshi et al., 2001). Two-pulse phase-modulated proton decoupling scheme (Bennett et al., 1995) was applied during free evolution and acquisition periods. The typical decoupling field strength was 84 kHz. The data were collected with an 8-ms evolution in the indirect dimension; 1434 complex *t*_2_ and 128 real *t*_1_ points with 2048 scans. A relaxation delay of 1.5 s was applied. Each spectrum was recorded over a period of ~140 h. Prior to Fourier transformation, the data were zero-filled to 4096 points, and an exponential apodization of 25 Hz was applied. ^13^C resonances were externally referenced with respect to backbone CO signal of solid glycine· HCl at 176.04 ppm on the TMS scale. The data were processed with Bruker Topspin 3.1 and further analyzed by using the Sparky 3.114 (Goddard and Kneller, 2008).

## Results

**Figure 2:**
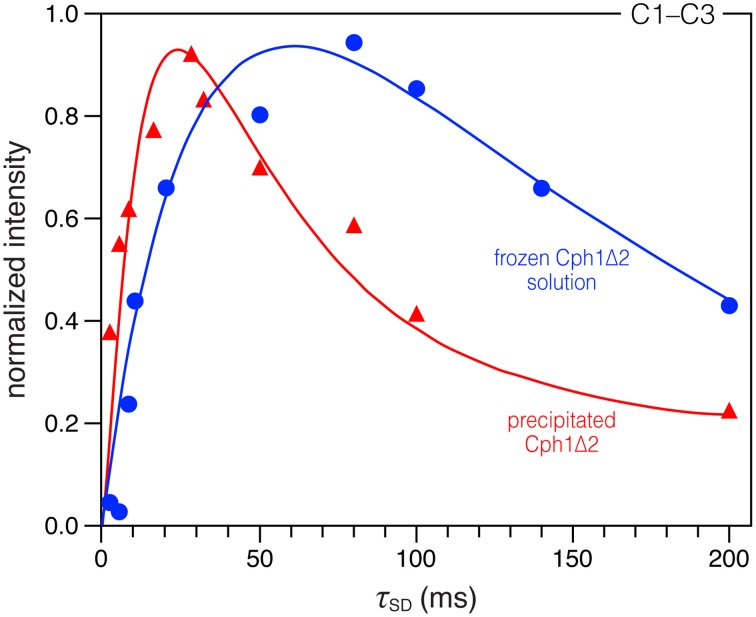
**Comparison of DARR build-up curves for the selected C1–C3 correlation in the precipitated and frozen solution states**. Spin-diffusion mixing times (τ_SD_) between 2 and 200 ms were used. All spectra were recorded under the similar conditions (with a MAS rate of 13 kHz at −50 °C, see Material and Methods for more details). The data were fitted by nonlinear least-square method (Ernst and Meier, 1998). For time evolution less than 200 ms, we expect the main contribution for the correlation intensities to be due to spin diffusion.

2D ^13^C–^13^C correlation spectra of the AmS-precipitated *u*-[^13^C,^15^N]-PCB-holoCph1Δ2 in its red-absorbing Pr form were recorded for ^13^C chemical shifts (δ^C^) of the bilin chromophore with two DARR mixing periods of 2 and 28 ms (Figure 1, red and purple, respectively; enlarged views of the spectra with external 1D projections along both dimensions shown in Figure S1). With a shorter mixing time of 2 ms, the DARR spectrum (red) is dominated by correlations occurring between strongly coupled (e.g., directly bonded) spins, whereas the data acquired with a 28-ms mixing time (purple) also reveal weak, through space ^13^C–^13^C couplings (e.g., long-range distances). It should be noted that the DARR experiments on this sample with mixing times beyond 40 ms yield much decreased overall signal intensity with many expected correlations only partially resolved (e.g., C1–C3 correlation build-up curve shown in Figure 2, red). Whereas, in the frozen solution states of canonical phytochromes a mixing time of 50 ms was found to be optimal for revealing the long-range ^13^C–^13^C correlations (Rohmer et al., 2008; Song et al., 2012). Thus, DARR mixing in the precipitated sample shows a much faster build-up behavior for long-range correlations relative to the frozen one, indicative of a more efficient spin-diffusion process between protons (Huster et al., 2002; Akbey et al., 2012).

**Figure 3:**
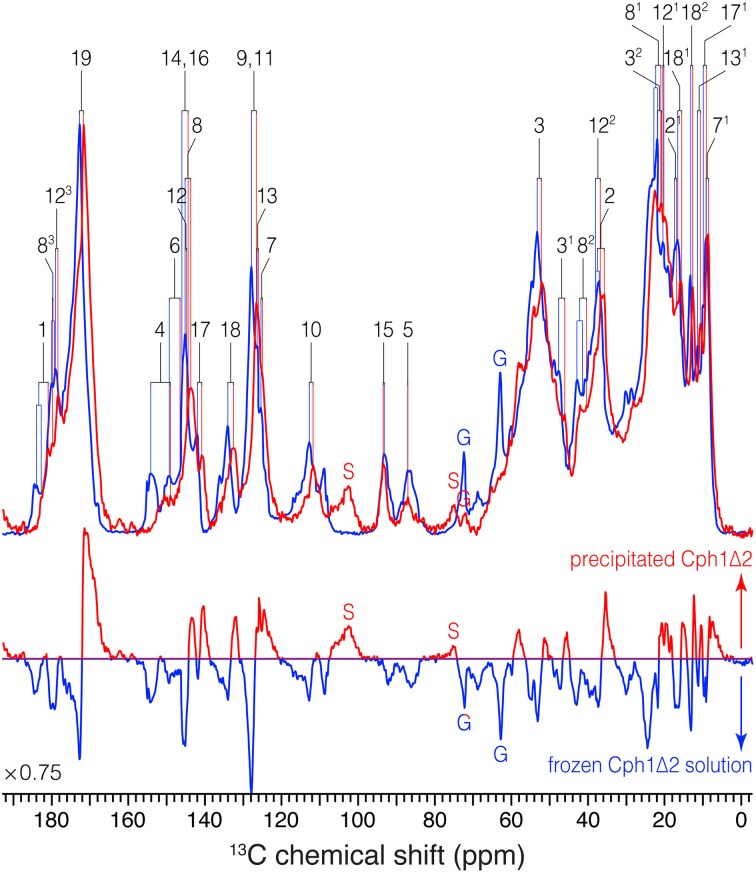
**1D ^13^C MAS spectra of *u*-[^13^C,^15^N]-PCB-Cph1Δ2 in the Pr state as an AmS pellet and as frozen solution**. ^13^C signals in the partial dehydrated (red) and frozen solution (blue) states are labeled. Signals of the natural abundance glycerol carbons at 62.8 and 72.1 ppm (Williamson et al., 2001; Rosay et al., 2002; Rohmer et al., 2008) are labeled as “G”. The spinning sidebands are labeled as “S”. Normalized difference spectrum (bottom) was calculated as spectrum from the precipitated sample minus that of frozen solution. The MAS rate of both experiments was maintained at 13 kHz. Typical ^1^H π/2 and ^13^C π pulses were 3.0 and 5.2 μs, respectively. ^13^C transverse magnetization created by the ramped CP was transformed from ^1^H with a contact time of 2.048 μs for both spectra. For ^1^H decoupling, two-pulse phase modulation scheme with a pulse duration of 5.5–7 μs and a ^1^H r.f. field strength of ~70 kHz were employed. Both spectra were recorded with 8 k scans with a recycle delay of 1.5 s. A Lorentzian apodization function with line broadening factors of 20 Hz was applied to the data processing.

The ^13^C assignment of two Pr spectra of the precipitated Cph1Δ2 (Figure 1) is based on our previous DARR data from its frozen solution (Rohmer et al., 2008): preliminary assignment achieved by analysis of direct correlations between ^13^C spins (red) and validated by indirect correlations originated from weak polarization transfers among isolated ^13^C spins (purple). For example in the precipitated Cph1Δ2, the previously unambiguous assignment for the propionate side-chains of two inner rings *B* and *C* in the frozen solution because of the signal overlapping of C8/C8^1^ and C12/C12^1^ (Rohmer et al., 2008) can be assigned unequivocally. Well-defined correlation networks of both propionates, C8 (144.2 ppm)–C8^1^ (21.0 ppm)–C8^2^ (40.4 ppm)–C8^3^ (179.8 ppm) and C12 (144.9 ppm)–C12^1^ (20.2 ppm)–C12^2^ (36.9 ppm)–C12^3^ (178.5 ppm) are apparent in the spectrum recorded with a mixing period of 2 ms (Figure 1, red). These assignments are confirmed by multi-bond correlation peaks involving non-propionate carbons such as C7 (125.2 ppm)/C7^1^ (8.7 ppm)–C8^1^/C8^2^/C8^3^, C10 (111.8 ppm)/C11 (126.5 ppm)–C12^1^, C13 (126.2 ppm)–C12^1^/C12^2^/C12^3^, and C13^1^ (10.7 ppm)–C12^2^/C12^3^ resolved in the 28-ms DARR mixing spectrum (Figure 1, purple). Intriguingly, for the *C*-ring propionate in the frozen solution state, two sets of chemical shifts were observed, indicative of local mobility of the chromophore and structural plasticity of the protein pocket (Song et al., 2011a), whereas only a single set was resolved for the precipitated Cph1Δ2. Similarly, no signal splitting in this sample was observed for its bilin ring *A* as a single correlation network for C1 (181. 5 ppm)–C2 (35.8 ppm)–[C2^1^ (16.9 ppm)]–C3 (52.2 ppm)–[C3^1^ (46.1 ppm)–C3^2^ (20.9 ppm)]–C4 (149.5 ppm). In general, the ^13^C MAS spectrum is better resolved in the solution state in terms of relatively narrow spectral lines which become broader on precipitation (see Figure 3 for comparison and discussed below).

**Figure 4:**
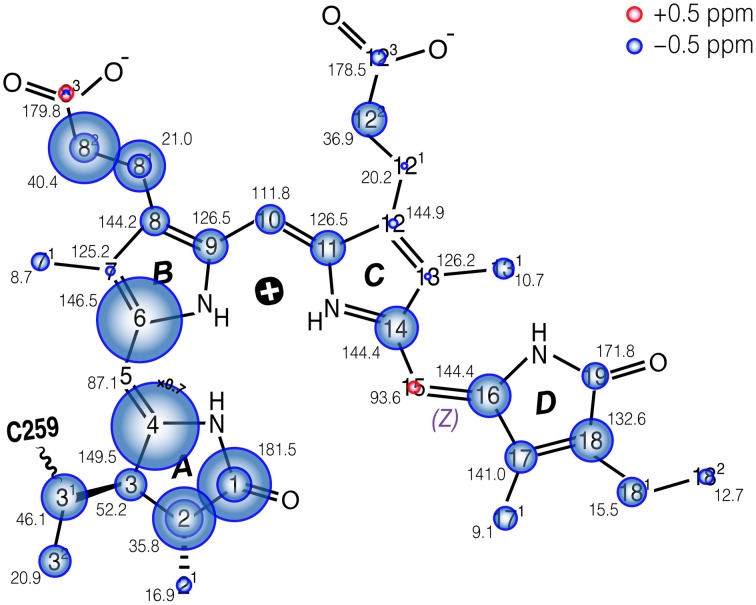
**Schematic of the changes in ^13^C shifts of the *u*-[^13^C,^15^N]-PCB chromophore in Cph1Δ2 in the Pr state as an AmS pellet and as frozen solution**. The size of the circles is proportional to the Δδ^C^ as AmS precipitate minus frozen solution. Carbons showing doublings as frozen solution (Rohmer et al., 2008) are labeled with two circles. δ^C^ values of AmS precipitate as Pr are labeled by black numbers. δ^C^ of both Cph1Δ2 Pr samples are summarized in Table S1.

The complete ^13^C bilin assignment in the precipitated Cph1Δ2 is summarized in Table S1, and the changes in its ^13^C shifts (Δδ^C^) upon precipitation is illustrated in Figure 4. The main features are as follows:

*(i)* Almost all the bilin signals move upfield, as represented by blue circles in Figure 4, amongst which most ^13^C atoms (27 of 33) exhibit moderate shifts of −0.5 ≤ Δδ^C^ ≤ −2.0 ppm (Table S1). The global Δδ^C^ induced by the partial dehydration reflect changes of the electronic structure of the bilin chromophore as well as the modification of its interactions with the protein surrounding. One might doubt whether the ^13^C chemical shift scale has shifted between these two experiments. However, in addition to standard external referencing procedure (see **Material and Methods**), both ^13^C MAS spectra show the same positions of glycerol natural abundance signal as internal standard (labeled as “G”, Figure 3). Also, the precision of ^13^C chemical shift measurement was controlled by comparing the spectra of frozen Cph1Δ2 solution sample obtained before (shown in Figure 3, blue) and after (data not shown) a series of measurements of precipitated sample, only subtle variations of the ^13^C chemical shifts were observed in the two spectra (before/after), and both are in line with the values reported (|Δδ^C^|≤ 0.2 ppm Rohmer et al., 2008). Hence, we conclude that the observed collective upfield shift is real and not an artifact that might have been occurred since wobbling of samples with high salt concentration is indeed difficult.
*(ii)* Δδ^C^ occurring in the region of pyrrole rings *A* and *B* are larger than those of rings *C* and *D*, in which C4 and C6 associated with the *A*–*B* methine bridge experience the largest upfield shifts of −4.4 and −3.0 ppm respectively. This likely reflects some conformational change of ring *A*, e.g., a different *A*-ring orientation relative to the *B*/*C*-ring plane. Also, the robust ^13^C shifts localized at bilin ring *A* and the propionate side-chain of ring *B* imply the modification of bilin–protein interactions because of a tight packing around rings *A*–*C* seen in the structures of Cph1 and plant phytochromes (Essen et al., 2008; Burgie et al., 2014).
*(iii)* The subtle shifts found at C9 and C11 (Δδ^C^ = −1.2 ppm) strongly suggest the bilin ring system in the precipitated sample retains the protonation state (Rohmer et al., 2010b), i.e., all four bilin nitrogens are fully protonated and thus positively charged, as in the frozen solution sample as both Pr and Pfr (Rohmer et al., 2008).
*(iv)* As can be seen from Figure 3, the ^13^C line broadening of the bilin signals occurs when protein is precipitated (expressed as full-width at half maximum, ν_1∕2_, summarized in Table S2). The broadening is most significant in the signals in/around *A*-ring region, for example, the ^13^C linewidth of the ethylidene side-chain C3^1^ increases from 342 Hz in the frozen solution to 489 Hz in the precipitated sample. A similar extent of line broadening is seen for C4 which broadens from 286 Hz (frozen solution) to 459 Hz (precipitate) and for C6 from 213 to 384 Hz. Also, a number of signals from rings *C* and *D* broaden dramatically like C12, C17, and C18 (Table S2). The observed increase in ^13^C linewidth upon precipitation does not result from the interference between ^1^H decoupling and methyl group motion at low temperatures (Maus et al., 1996). For optimal decoupling performance in the case of Cph1Δ2 precipitation, we scanned the ^1^H r.f. field in a wide range at −50 °C, as for the spectra shown in Figure 3 (red). Also, the proton exchange with water molecules seems unlikely to be relevant for the observed ^13^C line broadening which occurs not only in the positions close to hydrogen-bonding functions but spreads over the entire bilin chromophore (Table S2).

## Discussion

### The collective ^13^C upfield shift of bilin carbons

The most striking finding is the upfield shift of almost the entire bilin chromophore (as shown in Figure 4). This shift implies stronger shielding, most likely due to a reduced paramagnetic shift of the bilin carbons. Paramagnetic chemical shifts are related to the spatially-extended and non-spherical π-orbital structures of the ^13^C nuclei. Decay of ^13^C shifts can be related to a dense packing of the bilin chromophore in its binding pocket. This effect occurs in particular at rings *A* and *B*, suggesting that the change of packing is especially pronounced in this region. For example, major bilin δ^C^ changes are seen at C1 (−2.6 ppm), C2 (−2.2 ppm), and C3^1^ (−1.6 ppm) associated with the ring *A* (Figure 4). We suggest that these effects arise directly from a modification of the protein–chromophore linkage. Also, the C4 and C6 associated with the *A*–*B* methine bridge exhibit striking upfield shifts (−4.4 and −3.0 ppm respectively) on partial dehydration, reflecting a contortion of the thioether linkage which may also change the tilt of ring *A* in relation to the plane of rings *B* and *C*. Such deviations may arise from proximity of electrostatic charges and steric effects due to the tighter packing. This interpretation is also supported by the observed increase of spin-diffusion efficiency upon precipitation.

In presence of concentrated AmS, the effective size of the protein molecule decreases, together with increased hydrophobic protein–protein interactions resulting in a higher packing density in the partial dehydrated state primarily for the surface groups, and a shortening of the internuclear distances compared to those in the frozen solution (Kachalova et al., 1991). The strength of dipolar couplings (e.g., between ^1^H–^1^H and ^1^H–^13^C) in this state is thus increased because it is proportional to the inverse cube of the distances between them (Huster et al., 2002; Reichert et al., 2004; Diakova et al., 2007). In this study, we observe that such difference in packing density between the partial dehydrated and frozen solution states affects not only surface groups but also the bilin and its hydrophobic binding pocket too.

A more compact protein environment of the bilin on precipitation is supported by the faster ^13^C correlation build-up behavior observed in the DARR experiments. Taking C1–C3 correlation (181.5/52.2 ppm Figure S1) as an example, a sharp maximum is reached at mixing time of 28 ms (build-up curve shown in Figure 2, red), whereas in the solution state this correlation continues to increase up to 80 ms (Figure 2, blue). The build-up curve for C1–C3 correlation in Figure 2 also reveals differences between the two states at short mixing times around 2 ms. In the partially-dehydrated state (red), the correlation is already intense, whereas the correlation in the solution state (blue) is just above the *S*/*N* level. Similar build-up behaviors are also seen for most correlations involving pyrrolic carbons: a maximum intensity is reached after a short mixing period of 8–32 ms (build-up curves not shown). The faster build-up rates observed in the precipitated state indicate a more efficient spin diffusion than in the frozen solution state, thus facilitating the cross-talk between protons. Also, local dynamics leads to a reduction of the spin-diffusion rates constants. For the Cph1Δ2 precipitate, larger spin-diffusion rate constants can reasonably be expected because of the faster build-up behavior of the bilin correlations, suggesting a decrease in mobility within the bilin-binding pocket. Finally, although faster correlation build-up rates are observed in this sample, the maximum intensity reached is slightly smaller compared to the frozen solution (Figure 3). This could arise from relaxation effects and conformational transitions between different protein subconformations (energy minima), both of which are hydration-dependent (Zanotti et al., 1999; Krushelnitsky et al., 2009; Akbey et al., 2012). These data thus demonstrate that the process of partial dehydration causes changes of electronic structure of the bilin as well as its mobility within the pocket.

### An increased heterogeneous bilin environment

The Pr ground-state heterogeneity is common in Pr-state phytochromes including both canonical Cph1 (Sineshchekov et al., 2002; Rohmer et al., 2008; Mroginski et al., 2009; Mailliet et al., 2011; Song et al., 2011a, 2012; Kim et al., 2014a) and oat phyA3 (Schmidt et al., 1998; Song et al., 2012) as well as bacteriophytochromes from *Agrobacterium, D. radiodurans*, and *R. palustris* (von Stetten et al., 2008; Wagner et al., 2008; Toh et al., 2010). A heterogeneous ground-state population was also noted for phytochrome-related CBCRs such as NpR6012g4 (Kim et al., 2012a,b,c; Rockwell et al., 2012; Chang et al., 2013). Our previous NMR study on frozen Cph1Δ2 sample revealed the coexistence of two Pr isoforms in the solution, distinguished by their hydrogen-bonding networks and charge distribution patterns in the tetrapyrrole cavity (Song et al., 2011a). This idea has been extended by Kim et al. (2014a): they demonstrated using temperature-dependent pump–probe (PP) spectroscopy and singular-value decomposition (SVD) analysis that in solution the Pr subpopulations (fluorescent vs. photoactive) are in equilibrium at ambient temperatures and are associated with changes in the heat capacity (*C*_*p*_) of the protein.

For the precipitated Cph1Δ2, however, the ^13^C bilin signals are broader than those measured in frozen solution even for a number of peripheral carbons below ~50 ppm (Figure 3 and Table S2), suggesting a more heterogeneous protein environment of the bilin. Such a situation can arise from the amorphous character of the partial dehydrated sample (Kennedy and Bryant, 1990; Jia and Liu, 2006; Krushelnitsky et al., 2009), e.g., a broad distribution of protein subconformations with different interactions with the bilin (see below). The static disorder would be associated with the structural rearrangement of protein surface area because the addition of neutral salt like AmS constricts the hydration shells around the protein (Adamson and Gast, 1997) and thus results in increased hydrophobic protein–protein interactions. Although the possibility of structural distortions due to the formation of non-native electrostatic contacts between polar and charged groups in the partial dehydrated protein (Griebenow and Klibanov, 1995; Zanotti et al., 1999), the moderate ^13^C shifts of −0.5 ≤ Δδ^C^ ≤ − 2.0 ppm for most bilin carbons on partial dehydration (Table S1) would rule out major rearrangement of key protein–chromophore interactions. Moreover, water percolation through the protein interior is unlikely because buried water molecules are mainly strategically placed and tightly bound, and also because their population (of full hydration) is too low for them to form interconnected threads.

Intriguingly, C4 and C6 associated with the *A*–*B* methine bridge show not only the largest ^13^C chemical shift changes upon precipitation but also the greatest degree of line broadening (Tables S1,S2). A similar effect is also seen for other pyrrolic carbons associated with the ring *A* and its linkage to the protein. These obvious differences of direct protein environment of the ring *A* might reflect also changes of the water networks around the ring. Hence, under precipitation, the protein becomes more densely packed and its structural order is partially lost. These changes affect in particular rings *A* and *B*, here in addition also a conformational change of the chromophore, probably a change of the dihedral angle about the C4 = C5 bond occurs. Since the less affected rings *C* and *D* are more hidden in the protein interior, i.e., the ring *D* is completely shielded from the solvent by the side-chains of several residues of the GAF domain and the tongue region (Essen et al., 2008), it is reasonable to assume that changes in the water environment, caused by the partial dehydration, are responsible for these changes. It appears that the lack of nearby water molecules do not modify photoprocess affects between rings *C* and *D* but rather result in a fixation of the conformations. That implies that a fully-hydrated protein does not have a single conformation but a range of conformations which appear to be averaged on NMR timescale. Water may act as “lubricant” in protein conformational changes. As the sample is precipitated, less free water molecules are available, so the ordered protein conformation collapses, and thus protein molecules in different subconformations become trapped. It has been proposed from the Cph1Δ2 Pr crystal structure (PDB code 2VEA) that the bilin is sealed off from the solvent (Essen et al., 2008), although the higher-resolution structure of the Y263F mutant (PDB code 2ZQ5) implies that the seal is far from perfect (Mailliet et al., 2011). Future MAS NMR experiments might reveal which water molecules in the tetrapyrrole cavity (especially those in close contacts with the ring *A*) are affected in the process of partial dehydration, allowing their functional role in the reaction dynamics to be understood. It is evident from our MAS data that Cph1Δ2 precipitate as Pr shows increased heterogeneity of bilin environment.

## Conclusion

AmS precipitation is a mild, reversibly treatment commonly used to purify and concentrate proteins. It acts by withdrawing water from the hydration shell surrounding proteins, thereby reducing their solubility. The precipitate is quite dense and can be compacted by centrifugation, thereby offering a potential new route to gaining 3D structural information via solid-state NMR which otherwise requires highly-concentrated frozen samples. On the other hand, AmS precipitation might have more extensive effects, blocking access to the native structure. Here we have investigated the structure of AmS-precipitated holo-Cph1Δ2 *in vitro* assembled with *u*-[^13^C,^15^N]-PCB in its Pr state using solid-state NMR. This represents a useful case study as earlier studies showed that not only is the phytochrome photochromic state unaffected by AmS precipitation, resonance Raman spectra differ only slightly between AmS precipitates and frozen solutions (Matysik et al., 1995). We have extensively studied frozen solutions of Cph1Δ2 phytochrome using solid-state NMR, thus we are in a good position to assay possible effects of the AmS technique with Cph1Δ2. Indeed, the bilin ^13^C lines are broadened in the precipitated state, probably as a result of a more heterogeneous bilin environment. We identify a significant collective upfield ^13^C chemical shift of the bilin compared to the ^13^C data obtained from the frozen solution which reflect more dense sample packing around the bilin chromophore likely caused by the partial dehydration. Additionally, the MAS data reveal a dehydration-induced conformational change of the bilin chromophore, in particular for rings *A* and *B*. However, other key factors capable of modulating light absorption like protonation of the bilin and its direct hydrogen-bonding network seem to be unaffected by AmS precipitation. We conclude, therefore, that AmS precipitation can have significant effects throughout the protein and thus that the method, while perhaps useful in specific cases, might not provide *bona fide* structure/functional information. On the other hand, this method might allow for obtaining information on the local water pool and its exchange to the bulk. Future studies with AmS (or other salts) might elucidate how the salt-related dehydration process affects protein structure.

### Conflict of interest statement

The authors declare that the research was conducted in the absence of any commercial or financial relationships that could be construed as a potential conflict of interest.
