# Supplementary material for: NMR chemical shift pattern changed by ammonium sulfate precipitation in cyanobacterial phytochrome Cph1
Source: Front Mol Biosci. 2015 Jul 28;2:42. doi: 10.3389/fmolb.2015.00042 (PMC4516977; doi:10.3389/fmolb.2015.00042)
Supplement: Supplementary file 1 [file DataSheet1.PDF]

Supplementary Information for

NMR chemical shift pattern changed by ammonium sulfate precipitation in cyanobacterial phytochrome Cph1

Chen Song[1,2], Christina Lang[3], Jakub Kopycki[3], Jon Hughes[3], and Jörg Matysik[1,2]*

[1]*Leids Instituut voor Chemisch Onderzoek, Universiteit Leiden, 2300 RA Leiden, The Netherlands;*

[2]*Institut für Analytische Chemie, Universität Leipzig, Linnéstraße 3, D-04103 Leipzig, Germany;*

[3]*Institut für Pflanzenphysiologie, Justus-Liebig-Universität Gießen, Senckenbergstraße 3, D-35390 Gießen, Germany.*

*To whom correspondence should be addressed. E-mail: joerg.matysik@uni-leipzig.de.

This PDF file includes:

Figure S1

Tables S1 and S2

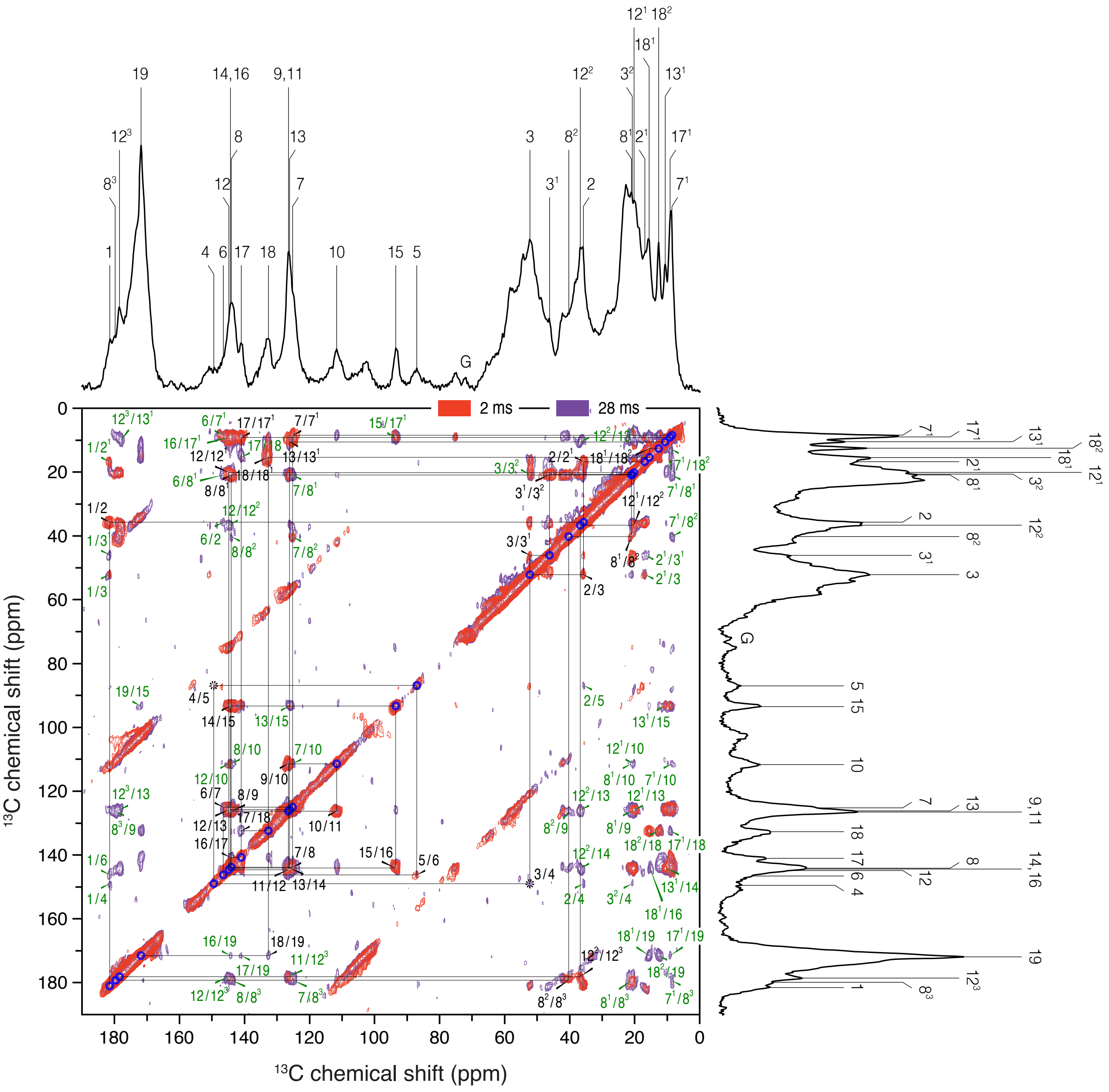

Figure S1 **Enlarged contour plots of the 2D ^{13}C–^{13}C DARR NMR spectra of *u*-[^{13}C,^{15}N]-PCB-Cph1Δ2 precipitated in AmS as Pr.** The spectra were recorded with two ^{13}C homonuclear recoupling mixing times of 2 (red) and 28 (purple) ms. Projections of the 2D spectra along ω_1-dimension (right trace) and ω_1-dimension (top trace) are displayed. Signals of the natural abundance glycerol carbons are labeled as “G”.

		Cph1Δ2 Pr		
PCB chromophore carbon		$\delta^{C}_{\text{frozen solution}}$ (ppm)‡	$\delta^{C}_{\text{AmS precipitate}}$ (ppm)§	$\Delta\delta^{C}_{\text{AmS precipitate – frozen solution}}$ (ppm)
ring A	**1**	182.7 (**1^a**)	181.5	**–1.2**
		184.1 (**1^b**)		**–2.6**
	**2**	37.1 (**2^a**)	35.8	**–1.3**
		38.0 (**2^b**)		**–2.2**
	**2^1**	17.4	16.9	**–0.5**
	**3**	53.3	52.2	**–1.1**
	**3^1**	47.6	46.0	**–1.6**
	**3^2**	22.0	20.9	**–1.1**
	**4**	153.9	149.5	**–4.4**
A–B	**5**	87.1	87.1	**0.0**
ring B	**6**	149.6	146.6	**–3.0**
	**7**	125.5	125.2	**–0.3**
	**7^1**	9.3	8.7	**–0.6**
	**8**	145.2	144.2	**–1.0**
	**8^1**	22.8 (**8^{1a}**)	21.0	**–1.8**
		21.8 (**8^{1b}**)		**–0.8**
	**8^2**	42.9 (**8^{2a}**)	40.4	**–2.5**
		41.4 (**8^{2b}**)		**–1.0**
	**8^3**	180.0 (**8^{3a}**)	179.8	**–0.2**
		179.3 (**8^{3b}**)		**+0.5**
	**9**	127.7	126.5	**–1.2**
B–C	**10**	112.8	111.8	**–1.0**
ring C	**11**	127.7	126.5	**–1.2**
	**12**	145.2	144.9	**–0.3**
	**12^1**	20.4	20.2	**–0.2**
	**12^2**	38.1	36.9	**–1.2**
	**12^3**	179.0	178.5	**–0.5**
	**13**	126.4	126.2	**–0.2**
	**13^1**	11.4	10.7	**–0.7**
	**14**	145.9	144.4	**–1.5**
C–D	**15**	93.2	93.6	**+0.4**
ring D	**16**	145.9	144.4	**–1.5**
	**17**	142.1	141.0	**–1.1**
	**17^1**	9.9	9.1	**–0.8**
	**18**	134.1	132.6	**–1.5**
	**18^1**	16.5	15.5	**–1.0**
	**18^2**	13.2	12.7	**–0.5**
	**19**	172.7	171.8	**–0.9**

‡Rohmer et al., 2008; §present work.

Table S1 **^{13}C chemical shifts of the *u*-[^{13}C,^{15}N]-PCB-Cph1Δ2 precipitated in AmS as Pr.** PCB δ^{C} values obtained from Cph1Δ2 as an AmS pellet are compared with those from a frozen solution sample. ^{13}C chemical shift differences ($\Delta\delta^{C}$) of PCB in the two samples were listed as AmS precipitate minus frozen solution. The PCB chromophore numbering is according to Figure 4.

		Cph1Δ2 Pr				
		**frozen solution**‡		**AmS precipitate**§		
PCB chromophore carbon		δ^C (ppm)	$\nu_{1/2}$ (FWHM, in Hz)	δ^C (ppm)	$\nu_{1/2}$ (FWHM, in Hz)	$\Delta\nu_{1/2}^{\text{AmS precipitate – frozen solution}}$ (Hz)
ring A	**1**	184.1	290	181.5	303	**+13**
	**2**	37.1	420	35.8	445	**+35**
	**2^1**	17.4	466	16.9	502	**+36**
	**3**	53.3	435	52.2	527	**+92**
	**3^1**	47.6	342	46.0	489	**+147**
	**3^2**	22.0	476	20.9	512	**+36**
	**4**	153.9	286	149.5	459	**+173**
A–B	**5**	87.1	411	87.1	529	**+118**
ring B	**6**	149.6	213	146.6	384	**+171**
	**7**	125.5	278	125.2	290	**+12**
	**7^1**	9.3	373	8.7	358	**–15**
	**8**	145.2	289	144.2	376	**+87**
	**8^1**	22.8	537	21.0	551	**+14**
	**8^2**	42.9	412	40.4	497	**+85**
	**8^3**	180.0	397	179.8	422	**+25**
	**9**	127.7	405	126.5	399	**–6**
B–C	**10**	112.8	359	111.8	443	**+84**
ring C	**11**	127.7	429	126.5	422	**–7**
	**12**	145.2	270	144.9	401	**+131**
	**12^1**	20.4	511	20.2	490	**–21**
	**12^2**	38.1	519	36.9	545	**+26**
	**12^3**	179.0	379	178.5	366	**–13**
	**13**	126.4	436	126.2	450	**+14**
	**13^1**	11.4	463	10.7	479	**+16**
	**14**	145.9	473	144.4	539	**+66**
C–D	**15**	93.2	426	93.6	397	**–29**
ring D	**16**	145.9	444	144.4	510	**+66**
	**17**	142.1	302	141.0	434	**+132**
	**17^1**	9.9	550	9.1	496	**–54**
	**18**	134.1	351	132.6	463	**+112**
	**18^1**	16.5	555	15.5	589	**+34**
	**18^2**	13.2	495	12.7	487	**–8**
	**19**	172.7	469	171.8	454	**–15**

‡Song et al., 2011b; §present work.

Table S2 **^{13}C FWHM (full-width at half maximum) changes of the PCB chromophore in the AmS-precipitated Cph1Δ2 as Pr.** ^{13}C FWHM values of PCB signals obtained from the Cph1Δ2 precipitation are compared with those from a routine frozen solution. ^{13}C FWHM changes ($\Delta\nu_{1/2}$) of PCB in the two samples were listed as AmS precipitate minus frozen solution. The PCB chromophore numbering is according to Figure 4.
